# A HISTORY OF ENVIRONMENTAL INJUSTICE: SEGREGATION AND AIR POLLUTION IN 1940 AND 2010 IN THE UNITED STATES

**DOI:** 10.1093/geroni/igae098.1754

**Published:** 2024-12-31

**Authors:** Jenni Shearston, Xiaorong Shan, Lucas Henneman, Taylor Mobley, Elizabeth Rose Mayeda, Rachel Morello-Frosch, Joan Casey

**Affiliations:** University of California Berkeley, Berkeley, California, United States; George Mason University, Fairfax, Virginia, United States; George Mason University, Fairfax, Virginia, United States; University of California Los Angeles, Los Angeles, California, United States; University of California Los Angeles, Los Angeles, California, United States; University of California Berkeley, Berkeley, California, United States; University of Washington, Seattle, Washington, United States

## Abstract

Air pollution is inequitably distributed across the United States, whereby minoritized racial/ethnic groups have greater exposure and consequent health effects. To understand present-day disparities, it is important to understand changes over time. We compared relationships between county-level air pollution and segregation in 1940 and present-day (2010). We calculated segregation as the Dissimilarity Index (Black vs non-Hispanic white population) using Census data. We evaluated three air pollution emission sources: oil/gas well locations (Enverus), fossil fuel powerplant locations (Energy Information Administration, Environmental Protection Agency), and gasoline consumption (proxy for traffic-related air pollution; Federal Highway Administration). We estimated rurality-stratified associations between each segregation/pollution pair by year with non-linear generalized additive models, adjusted for population density and within-state clustering. Mean county-level segregation decreased from 1940 to 2010 for rural (-33%) and urban (-42%) counties. In 1940, we found more oil/gas wells and lower gasoline consumption in rural counties with higher segregation (>median). For example, if segregation increased from the median to the 75th percentile, we observed a 0.15 kg/year decrease (95% confidence interval: -0.23, -0.07) in gasoline consumption. We saw no association between segregation and powerplants in rural 1940 counties, but observed fewer powerplants and lower gasoline consumption in counties with less segregation (< median) in 2010. In 1940, we found fewer oil/gas wells in urban counties with higher segregation. We found suggestive evidence of a relationship between segregation and air pollution in 1940 and 2010, with substantial differences by rurality. Policy to reduce air pollution should consider historical exposure inequities in emission sources.

